# From literacy to learning: The sequential mediation of attitudes and enjoyment in AI-assisted EFL education

**DOI:** 10.1016/j.heliyon.2024.e37158

**Published:** 2024-08-30

**Authors:** Jiqun Fan, Qinqing Zhang

**Affiliations:** School of Foreign Languages, Huainan Normal University, Huainan, Anhui Province, 232001, China

**Keywords:** AI-Assisted EFL learning, Continuance intention, AI literacy, Foreign language enjoyment, Learner attitudes

## Abstract

Artificial intelligence (AI) has become increasingly prominent in English as a Foreign Language (EFL) education. However, research on the factors influencing learners' sustained engagement with AI-assisted learning is scarce. This study bridges this gap by investigating the impact of AI literacy, learner attitudes toward AI-assisted learning, and foreign language enjoyment (FLE) on Chinese EFL learners' continuance intention (CI) for AI-assisted EFL learning. A survey of 417 EFL learners was conducted, and structural equation modeling was utilized to elucidate the relationships between these variables. The findings reveal that AI literacy, learner attitudes, and FLE significantly predict the CI. Moreover, AI literacy indirectly influences CI by mediating learner attitudes and FLE. Finally, FLE mediates the relationship between learner attitudes and CI. The study verifies a sequential mediation model, providing new insights into EFL learners' cognitive and affective traits and extending current models on the continuance intention in AI-assisted EFL learning. The research underscores the necessity for educators and technology developers to consider learners' interests and feedback, which are crucial for the sustainable progress of AI-assisted EFL learning.

## 1 Introduction

Over the past three decades, integrating artificial intelligence (AI) with English as a Foreign language (EFL) learning has seen remarkable progress. This advancement has manifested in various domains, including automated question-answering systems, conversational agents (chatbots), machine translation, corpus development, intelligent tutoring systems, and sophisticated assessment mechanisms [1]. Despite these strides, the implementation of AI in EFL education remains nascent, and AI tools still exhibit gaps between the available technology and practice [2]. Furthermore, the exponential pace of AI development and the rapid turnover of technology generations may lead to information overload and the negative spillover effect, precipitating a discontinuation of use or a propensity for learners to frequently switch between AI products [3,4]. Learners' persistence in utilizing AI-assisted language learning tools is pivotal for this educational paradigm's sustainable and robust evolution. Nonetheless, the extant research probing the determinants that influence the sustained engagement with AI in EFL learning is far from sufficient [5], indicating a gap that warrants further scholarly attention.

Proficient use of technology in education requires a clear understanding and comprehensive knowledge of the technology itself. As information technology continues to evolve, learners' information technology literacy has undergone a dynamic historical evolution, progressing from technological literacy, information literacy, data literacy, media literacy, digital literacy, and eventually refining to AI literacy [6,7]. As a specialized aspect of digital literacy, AI literacy focuses on understanding AI technologies and their impact on information processing [8,9]. A prevalent definition characterizes AI literacy as the capacity of individuals to critically assess AI technologies, effectively utilize them in both virtual and real-world contexts, and engage in meaningful communication and collaboration with AI systems [10]. This definition encapsulates the essence of AI literacy as it pertains to the application of AI, aligning with the Technology Acceptance Model (TAM) framework that emphasizes perceived usefulness and ease of use [11]. Due to its short span of development, research into AI literacy remains less abundant compared to studies on literacy in earlier technological eras. The influence of AI literacy on the sustained use of AI technologies in EFL learning, and the underlying mechanisms at play, remain underexplored and thus merit further scholarly exploration.

In the extant research on the continued learning intentions associated with educational technology, theoretical models such as the Technology Acceptance Model (TAM) and the Theory of Planned Behavior (TPB) consistently identify learner attitude as a pivotal determinant of sustained engagement with technology [12]. As the application of Artificial Intelligence in Education (AIED) expands across diverse scenarios, the competition among AIED products has intensified, making learner attitude a crucial factor for the sustainable development of these products. Concurrently, the emotional dynamics of individual learners during the learning process are intrinsically linked to their attitudes. While studies on emotions in foreign language learning have gained momentum, their integration into AI-assisted learning scenarios is still under-researched [13].

AI education advocates for personalized learning experiences. Therefore, individuals' cognitive, attitudinal, and emotional factors during the learning process should be positioned at the forefront of AIED research [14]. In light of the current state of research, this paper proposes a predictive model for the continued intention for AI-assisted EFL learning, grounded in TAM and incorporating foreign language enjoyment. The study explores the mechanisms and boundaries within which AI literacy influences the sustained intention to engage with AI-assisted EFL learning technologies. It examines the mediating roles of attitudes toward AI-assisted EFL learning and foreign language learning. By doing so, this research extends the application of the TAM to the context of AI-assisted EFL learning, delving into the role of learners' positive emotions. The findings provide insights and a reference framework for the healthy development of AI-assisted EFL learning.

## 2 Literature review

### 2.1 Technology Acceptance Model and positive psychology

The Technology Acceptance Model was initially proposed by Davis and became an effective model for predicting user acceptance of information systems [15]. The theory is made up of core variables including perceived ease of use, perceived usefulness, attitude, and behavioral intention. According to TAM, perceived ease of use and perceived usefulness determine attitudes towards behavioral intention, which further influences actual use. Therefore, TAM is efficient and convenient in understanding individuals' general views towards new technology [16]. However, TAM has been criticized for only focusing on technological features and neglecting some other determinants [17]. Researchers often borrow variables from other theories and extend the TAM to offset the imbalance. For instance, in the realm of technology-assisted learning, scholars in their separate studies incorporated other variables in light of environmental and motivational characteristics and extended the model [[18], [19], [20]]. Despite the shortcomings, when it comes to AI-assisted EFL education research, TAM has still been widely used to explain learners’ intentions and behavior [[21], [22], [23]].

Positive psychology, introduced by Martin Seligman in 1988, aims to assist individuals in cultivating a sense of well-being and in achieving a proactive and meaningful state of living [24]. The theory is primarily composed of three interrelated and mutually reinforcing components: positive individual traits, positive subjective experiences, and positive institutions [25]. Since its inception, positive psychology has been extensively applied to the field of language learning, resulting in influential theoretical outcomes such as the "good language learner model" [26], the "social educational model for research on second language learning motivation" [27], and the "second language self-system" [28]. While positive psychology has been widely applied in EFL teaching research, research exploring learners' positive foreign language emotions in AI educational contexts remains scarce. There is a notable absence of empirical studies examining the extent to which learners' positive learning emotions influence their acceptance and recognition of AI technology in EFL learning.

### 2.2 EFL learners’ AI literacy and their attitudes toward AI-assisted learning

The attitude toward AI-assisted EFL learning technology represents the comprehensive evaluation that learners make regarding AI technology and its assistance in the EFL learning process, which mainly includes acceptance, understanding, and expectations of knowledge, technology, application, and even governance [29]. Learners' attitudes towards information technology and its use are closely related to their usage experience and are primarily divided into cognitive and affective aspects [30,31]. An individual learner's attitude towards information technology is directly proportional to their level of understanding and experience with the technology and inversely proportional to their level of fear towards it [32]. After learners acquire AI literacy, they may intend to accept AI applications [12]. It can be inferred that the higher the user's AI literacy, the deeper their understanding and recognition of the human-AI relationship. Therefore, this study proposes the following hypothesis.

H1 EFL learners' artificial intelligence literacy positively influences their attitudes towards AI-assisted EFL learning.

### 2.3 EFL learners’ AI literacy and foreign language enjoyment

AI literacy reflects an individual's cognition and confidence in artificial intelligence technology and its applications [33]. Despite the current dearth of literature supporting a relationship between AI literacy and foreign language enjoyment, previous studies have confirmed that digital literacy, which encompasses AI literacy, positively influences learners' positive emotions. For example, after surveying 7973 junior high school students from 214 schools in the Taiwan region, She et al. found that the higher the students' scientific literacy, the higher their positive cognitive level towards science and the higher their pleasant experience in the process of acquiring scientific knowledge [34]. Similarly, in an empirical investigation of 987 Chinese university EFL learners, Feng found that digital literacy could predict about 95 % of changes in the participants' foreign language enjoyment [35]. Therefore, college students' AI literacy is positively correlated with their sense of efficacy, resilience, and other positive emotions in foreign language learning. Based on this, the present study proposes the following hypothesis.

H2 EFL learners' AI literacy positively influences their foreign language enjoyment.

### 2.4 EFL learners’ attitudes toward AI-assisted EFL learning and foreign language enjoyment

Learning attitude is an important factor related to students' academic achievement [36], which can generate an individual's positive learning emotions. Even their attitude toward school can affect their academic enjoyment [37]. As technology-supported learning becomes widely accepted, the role of learning attitude towards technology has aroused scholarly attention. The learners' attitude toward the technology used in learning largely impacts the pleasure they can perceive in learning. For instance, in an empirical study of game-based learning, Baek & Touati stated that a player's attitude toward games predicted intrinsic motivation, the only variable in the study to predict game enjoyment [38]. Later, they constructed a model showing that learners' attitude in game-based learning predicts the enjoyment they can expect in the learning process [39]. However, in the context of AI-assisted learning, the related research is scant. In a recent study focused on AI-assisted EFL writing, Stornaiuolo et al. claimed that frequent exposure to such technology among young people can help form a positive evaluation, and thus generate a sense of pleasure from collaborative learning [40]. Based on the above analysis, this study proposes the following hypothesis.

H3 EFL learners' attitudes towards AI-assisted EFL learning positively influence their foreign language enjoyment.

### 2.5 EFL learners’ attitudes toward AI-assisted learning and their continuance intention for AI-assisted learning

Previous studies have indicated that a positive attitude towards technology is an important prerequisite for individuals to initially use or continue using technology [15,41]. Kwak et al. have empirically demonstrated that the attitude toward the use of AI technology positively influences the behavioral intentions of nursing students [42]. Centeno-Martín et al. found that gender, age, and attitude towards AI technology are the main determinants of the willingness to use AI technology [32]. The higher the attitude an individual learner holds towards AI technology, the stronger the willingness to use it turns out to be [43]. Dai et al. also surveyed Chinese university students on the continuance intention of MOOC learning, and identified attitude as a dominant factor influencing learners' continuance intention [44]. Based on this, the present study proposes the following hypothesis.

H4 EFL learners' attitudes towards AI-assisted EFL learning positively influences their AI-assisted learning continuance intention.

### 2.6 Learners’ language enjoyment and their continuance intention for AI-assisted EFL learning

FLE is the positive emotion that individuals experience after overcoming difficulties and completing learning tasks in the process of foreign language learning, thereby satisfying psychological needs [45]. Extant literature has shown that positive psychological emotions have a significant positive impact on the learner's intention to continue learning in different teaching scenarios such as offline, blended, and online [[46], [47], [48]]. For example, positive emotions in multimedia-based learning facilitate cognitive processes and learning and therefore retain the learner's sustained interest in the learning task [49]. In the context of mobile learning, positive anticipated emotions are found to reinforce the positive link between attitude towards mobile learning and desire to take mobile learning [50]. Also, the study by Shang & Lyv(2024) confirmed the influence of learners' positive emotions on their continuance intention within the context of MOOCs [51]. Based on this, the present study proposes the following hypothesis.

H5 EFL learners' FLE positively influences their AI-assisted learning continuance intention.

### 2.7 EFL learners ’AI literacy and their continuance intention for AI-assisted learning

Through empirical research, Mohammadyari & Singh found that learners' digital literacy significantly and positively affects their intention to continue using information technology for learning [52]. Therefore, digital literacy is identified as a key determinant in learners' continued acceptance of digital technology [53]. In the realm of AIED, AI literacy directly impacts perceptions of the use of AI for social good and learners’ behavioral intention for AI-assisted learning [54]. At the same time, previous studies have also found that literacy can influence individuals' use intention through variables including emotional and psychological factors [55,56], and attitude and enjoyment are common mediating variables for behavioral intentions [57,58]. Based on these, the present study proposes the following hypotheses.

H6 EFL learners' AI literacy positively influences their continuance intention for AI-assisted learning.

H7 EFL learners' ATT and FLE play a sequential mediating role on the relationship between learners' AI literacy and their continuance intention for AI-assisted learning.

H8 EFL learners' ATT mediates the relationship between learners' AI literacy and their continuance intention for AI-assisted learning.

H9 EFL learners' FLE mediates the relationship between learners' AI literacy and their continuance intention for AI-assisted learning.

**Fig. 1:**
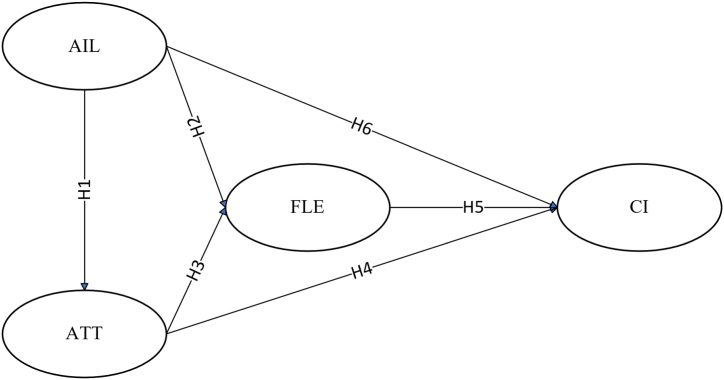
Hypothesized model.

Previous studies on the continuance intention of EFL learning have largely focused on online learning contexts, with relatively less attention given to scenarios of AI-assisted learning [[59], [60], [61]]. These studies tend to concentrate on the acceptance and application of technology, focusing on objective factors within learning activities, and paying insufficient attention to the learners' subjective factors, particularly emotional and attitudinal psychological aspects [62]. To fill the gaps, based on the above analysis, this study constructs a model of willingness to continue EFL learning with AI assistance and tests the mediation effect of EFL learners’ attitudes and foreign language enjoyment in the learning process (see Fig. 1).

## 3 Methods

### 3.1 Participants, context, and procedure

This study randomly selected 450 EFL students from three ordinary universities in a central province of China and distributed questionnaires in paper form. The aforementioned universities are promoting smart teaching in the reform of college English education, and teachers encourage students to use AI tools in EFL teaching. Students in the classroom were permitted to utilize AI software through personal smartphones and classroom desktop computers, receiving assistance from the AI software in areas such as word meaning lookup, corpus generation, automatic translation, and speech recognition. After the survey, 435 questionnaires were collected, of which 417 were valid. Further demographic information is shown in Table 1.

**Table 1:** Demographic information of the participants.

Demographic Information category	N	%
Gender		
Male	143	34.3
Female	274	65.7
Grade		
Freshmen	142	34.1
Sophomore	87	20.9
Junior	147	35.3
Senior	41	9.8
Age		
≤19	112	26.9
20	110	26.4
21	135	32.4
≥22	60	14.4
Academic major category		
Natural science disciplines	88	21.1
Engineering and technical disciplines	104	24.9
Social science disciplines	225	54.0
Total	417	

The paper questionnaires were distributed to students by the course instructor, who informed the students of the purpose of the research, promised to keep their answers confidential, and affirmed that the respondents could withdraw from the survey at any time. Students were also asked to read the questions carefully. After completing the survey, they were given a packet of instant coffee as a gift.

### 3.2 Measures

In this research, the latent variables examined encompass AI literacy(AIL), foreign language enjoyment (FLE), learners' attitudes towards AI-assisted EFL learning(ATT), and learners’ continuance intention for AI-assisted EFl learning(CI). Each item is evaluated using a 5-point Likert scale, where 1 signifies "strongly disagree" and 5 indicates "strongly agree."

The AI literacy dimension was measured using the AI literacy scale developed by Chai et al. [63]. This scale comprises six dimensions (e.g., "I know the processes through which deep learning enables AI to perform voice recognition tasks."). It reflects foreign language learners' understanding of the cognitive and basic principles of AI-assisted foreign language learning technologies. In this study, the scale's Cronbach's α value was 0.864, indicating good internal consistency. The fit indices were χ^2^/df = 2.829, GFI = 0.980, AGFI = 0.954, CFI = 0.984, TLI = 0.973, and RMSEA = 0.066, all of which are within acceptable ranges, demonstrating that the scale is both reasonable and reliable.

The dimension of FLE was measured using the Foreign Language Enjoyment Scale by Jiang & Dewaele [64]. This scale includes 10 items, such as "It is cool to learn English with AI assistance." In this study, the scale's Cronbach's α value was 0.942, indicating high internal consistency. The fit indices were χ^2^/df = 1.835, GFI = 0.971, AGFI = 0.955, CFI = 0.990, TLI = 0.987, and RMSEA = 0.045, showing good model fit and suitability for research.

To investigate learners' attitudes toward using AI technology in EFL learning, the study employed the Attitude Scale by Compeau and Higgins (1995) with slight modifications [65]. This dimension comprises five items, such as "AI-assisted EFL learning is a joyful experience." In this study, the scale's Cronbach's α value was 0.853, with fit indices of χ^2^/df = 2.759, GFI = 0.987, AGFI = 0.961, CFI = 0.989, TLI = 0.979, and RMSEA = 0.065, indicating good fit and reliability.

The continuance intention for AI-assisted EFL learning was measured using the Continuance Intention Scale by Mahmoud et al. [66]. This dimension includes four items, such as "If possible, I will continue with AI-assisted EFL learning." In this study, the scale's Cronbach's α value was 0.905, with fit indices of χ^2^/df = 1.774, GFI = 0.996, AGFI = 0.979, CFI = 0.999, TLI = 0.996, and RMSEA = 0.043, demonstrating that the scale is reasonable and reliable.

The overall KMO value of the scales in this study was 0.948, greater than 0.7, indicating suitability for factor analysis. Additionally, the *p*-value was less than 0.05, reaching a significant level, which confirms that the scales have ideal reliability and validity.

### 3.3 Data analysis

In this study, the analytical tools SPSS 26.0 and AMOS 24.0 were employed for the examination of data. Initially, a Harman single-factor test was employed to preemptively address the issue of common method bias, inherent in the self-reported nature of the survey data. Following this, descriptive and correlational analyses were executed to establish a foundational understanding of the dataset. The research then advanced to the evaluation of the proposed theoretical model via regression analysis. The culmination of this analytical sequence involved an exploration of mediation effects, facilitated by a bootstrapping technique that enabled the precise calculation of a bias-corrected 95 % confidence interval.

## 4 Results

### 4.1 Common method bias analysis

To ensure that the results of the questionnaire survey are not affected by common method bias, this study employed the Harman single-factor test method [67]. Before factor loading rotation, there were four factors with initial eigenvalues greater than 1. The first factor accounts for 42.63 % of the variance, which is less than the critical threshold of 50 % [68]. This met the measurement criteria and indicated that the study could proceed.

### 4.2 Measurement model analysis

This study utilized SPSS 26.0 to analyze the reliability and validity of the measurement scales. Using the eigenvalue method, four common factors were extracted, with each factor loading above 0.6, indicating that the questionnaire measures adequately reflect the four dimensions. The Cronbach's α values for each dimension were all greater than 0.7, demonstrating good internal consistency among the items within each dimension. Additionally, the composite reliability (CR) and average variance extracted (AVE) for each dimension were greater than 0.7 and 0.5, respectively (see Table 2). The square root of the AVE for each dimension was greater than the correlation coefficients with other dimensions (see Table 3), indicating that the model has good discriminant validity.

**Table 2:** Factor analysis, convergent reliability, and validity.

Constructs	Items	Estimate	S．E．	Z value	*p*	Factor Loading	α	C.R.	AVE
FLE	FLE01	1.000				0.861	0.942	0.943	0.625
	FLE02	0.796	0.043	18.311	***	0.741			
	FLE03	0.890	0.042	21.001	***	0.807			
	FLE04	0.939	0.045	20.830	***	0.803			
	FLE05	0.866	0.046	18.863	***	0.756			
	FLE06	0.889	0.044	20.289	***	0.791			
	FLE07	0.777	0.046	16.843	***	0.701			
	FLE08	0.847	0.046	18.456	***	0.745			
	FLE09	0.864	0.043	20.278	***	0.790			
	FLE10	0.905	0.036	25.258	***	0.892			
AIL	AIL1	1.000				0.686	0.864	0.864	0.516
	AIL2	1.024	0.080	12.727	***	0.701			
	AIL3	0.937	0.077	12.200	***	0.669			
	AIL4	1.035	0.077	13.390	***	0.743			
	AIL5	1.228	0.084	14.546	***	0.823			
	AIL6	0.918	0.075	12.312	***	0.676			
ATT	ATT1	1.000				0.799	0.853	0.854	0.539
	ATT2	0.837	0.060	14.063	***	0.680			
	ATT3	0.931	0.062	15.059	***	0.721			
	ATT4	0.839	0.057	14.843	***	0.712			
	ATT5	0.932	0.059	15.840	***	0.754			
CI	CI1	1.000				0.890	0.905	0.907	0.709
	CI2	0.869	0.043	20.306	***	0.790			
	CI3	0.921	0.041	22.398	***	0.836			
	CI4	0.915	0.040	23.087	***	0.850			

Note: *** indicates significance at the *p* < 0.001 level. AIL stands for artificial intelligence literacy; ATT for attitude toward AI-assisted EFL learning; FLE for foreign language enjoyment; and CI for continuance intention for AI-assisted EFL learning.

**Table 3:** Correlation metrics and discriminant validity.

Constructs	AIL	ATT	FLE	CI
AIL	**0.718**			
ATT	0.377	**0.734**		
FLE	0.436	0.697	**0.791**	
CI	0.410	0.600	0.637	**0.842**

Note: The lower triangle represents the correlation coefficients between dimensions, and the diagonal line in bold displays the square root of the AVE values.

### 4.3 Test for structural model

**Fig. 2:**
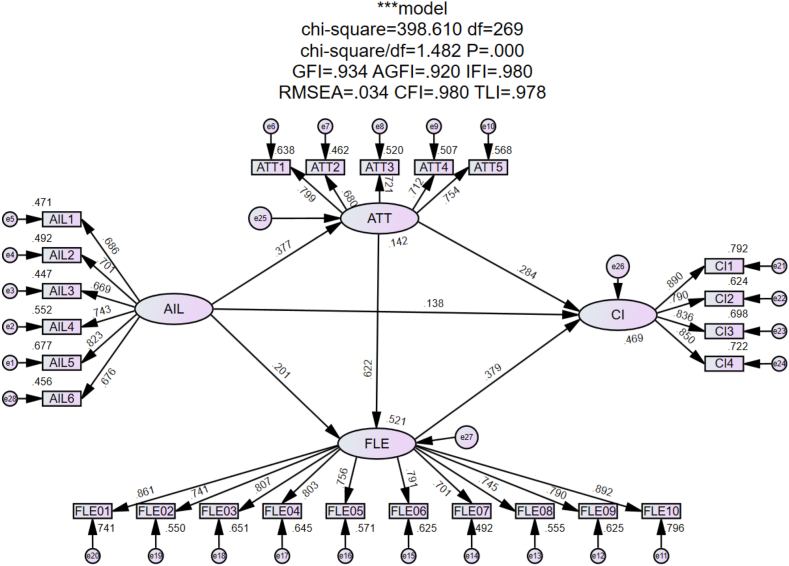
Strcutural model.

This study employed AMOS 24.0 to conduct a goodness-of-fit analysis of the theoretical research model. Following Kline's recommendations [69], the ideal model fit indices are χ^2^/df < 3, and CFI, GFI, IFI, AGFI, and TLI ≥0.90, while RMSEA and SRMR ≤0.08. In this study, χ^2^ = 398.610 with df = 269, resulting in χ^2^/df = 1.482. The goodness-of-fit indices were GFI = 0.934, AGFI = 0.920, CFI = 0.980, TLI = 0.978, RMSEA = 0.034, and SRMR = 0.031(see Table 4). These results indicated a good fit between the data and the model, allowing for further empirical analysis(see Fig. 2).

**Table 4:** Results of model fit indices.

Indices	*χ*^*2*^*/df*	*SRMR*	*RMSEA*	*GFI*	*AGFI*	*IFI*	*CFI*	*TLI*
Recommended values	<3.000	<0.080	<0.080	>0.900	>0.900	>0.900	>0.900	>0.900
Observed values	1.482	0.031	0.034	0.934	0.920	0.980	0.980	0.978

As shown in Table 5, the standardized path coefficients for Hypotheses H1-H6 were 0.377, 0.201, 0.622, 0.284, 0.379, and 0.138, respectively, with p-values all below 0.05, indicating that all six direct hypotheses were supported. The data suggested that artificial intelligence literacy (AIL) positively influences attitudes toward AI-assisted EFL learning (ATT), foreign language enjoyment (FLE), and continuance intention for AI-assisted EFL learning (CI), with the greatest effect observed on ATT. Additionally, ATT has a positive impact on both FLE and CI, and CI is also positively affected by FLE.

**Table 5:** Path analysis.

hypothesis	Pathways	B	SE	Z	*p*	β	Hypotheses
H1	AIL→ATT	0.415	0.065	6.407	***	0.377	Supported
H2	AIL→FLE	0.253	0.058	4.326	***	0.201	Supported
H3	ATT→FLE	0.708	0.061	11.58	***	0.622	Supported
H4	ATT→CI	0.279	0.065	4.271	***	0.284	Supported
H5	FLE→CI	0.328	0.057	5.781	***	0.379	Supported
H6	AIL→CI	0.150	0.053	2.825	0.005	0.138	Supported

Note: *** indicates significance at the *p* < 0.001 level.

### 4.4 Mediation analysis

As the results suggest, the direct effect path (AIL→CI) in the model proposed by this study was significant, indicating that the mediating path exhibits partial mediation. Significant tests of the mediating effect between AI literacy and the continuance intention for EFL learning with AI assistance were conducted using Amos 24.0 software. Non-parametric Bootstrap method was employed to examine the mediating effects of attitude toward AI-assisted EFL learning and foreign language enjoyment. The total effect value between AIL and CI was measured as 0.445, with a total mediating effect of 0.295. Both ATT and FLE showed significant mediating effects. The mediating paths "AIL→ATT → FLE→ CI ″, "AIL→ATT →CI ″, and "AIL→FLE →CI" all had confidence intervals after bias correction that did not include 0, and their *p*-values were all less than 0.05, indicating significant mediating effects. There were no significant differences among the various paths. The path "AIL→ ATT→CI ″ had the largest proportion in the total indirect effect, accounting for 39.3 % (see Table 6).

**Table 6:** Mediation analysis.

Path	β			Bootstrap bias-corrected 95 % CI		
		SE	Z-value	LLCI	ULCI	*p*
H7:AIL→ATT→FLE→CI	0.096	0.025	3.840	0.056	0.162	0.000
H8:AIL→ATT→CI	0.116	0.043	2.698	0.049	0.219	0.000
H9: AIL→FLE→CI	0.083	0.031	2.677	0.032	0.155	0.000
Total Mediation	0.295	0.048	6.146	0.207	0.398	0.000
Direct Effect	0.150	0.06	2.500	0.035	0.273	0.011
Total Effect	0.445	0.072	6.181	0.313	0.595	0.000

## 5 Discussion

This study, starting with learners' AI literacy, regarded learners' attitudes toward AI-assisted EFL learning and foreign language enjoyment as mediating variables, and explored the intrinsic mechanism of the impact of learners' AI literacy on the continuance intention to learn with AI assistance. The results showed that AIL, ATT, and FLE all have positive and significant impacts on CI. FLE exerts the strongest predictive role, echoing Weber & Harzer's finding that enjoyment can motivate learners to increase their learning investment and achieve better academic outcomes, which becomes an internal motivation and a compelling reason for continued learning [70]. Despite the widespread academic recognition of the positive impact of foreign language enjoyment on academic achievement [[71], [72], [73]], previous research has rarely integrated the concept of foreign language enjoyment into the study of continued learning intention, neglecting the positive influence of individual characteristics. This study, however, underscored the pivotal role of learners' positive emotions in AI-assisted EFL learning environments, as demonstrated by empirical findings. Meanwhile, according to the present study, learners' AI literacy plays the weakest role. This concurs with Lim's view that the cognitive awareness of AI educational applications is just the starting point, and learners' attitudes toward AI technology applications and their experiences in learning practice are crucial, serving as the ultimate goal of AI-enabled education services [74].

The present study also revealed that learners' attitudes toward AI-assisted EFL learning and foreign language enjoyment mediate the relationship between learners' AI literacy and their continuance intention to learn with AI assistance. Prior research on technology-empowered foreign language learning has predominantly concentrated on external factors such as course quality, ease of use and usefulness of learning platforms, technological proficiency, teacher-student interaction, and social environment [[75], [76], [77]]. In contrast to these, the present study placed a significant emphasis on the importance of individual emotions and psychological attitudes. Therefore, AI technology empowering EFL learning should focus on building immersive learning environments that integrate virtual and real experiences, aiming to create immersive, authentic, spatiotemporally strong, and collaboratively interactive language learning and cultural communication spaces [78,79].

Among the three indirect effect paths, “AIL → ATT → CI” registered the largest effect value, reflecting the crucial impact of learners' attitudes on their willingness to continue using AI for language learning. It also complies with previous findings that attitudes towards learning are closely related to the achievement of learning goals and the effectiveness of learning outcomes, serving as the belief support and motivational safeguard for learners [80,81]. This finding confirms the applicability of the TAM model to this study and further affirms the importance of learners' attitudes in EFL education research within AI contexts.

The study provided several insights into the practice of AI-assisted EFL teaching. Firstly, as the main body of foreign language teaching, higher education institutions should respect the intrinsic laws of literacy, explore the establishment of corresponding courses or modules, and foster trust and confidence among students and staff in AI technology usage [82]. Secondly, the positive emotions of learners in AI-assisted EFL learning should not be overlooked. This requires educational administrators and practitioners to heed students' positive experiences to effectively promote the continuous application of AI in EFL education scenarios [83]. Thirdly, the research suggested a shift from technology-centered to human-centered, emphasizing the cultivation of literacy that focuses on people and transcends the technical obstacles [84], and striving to develop students' multidimensional capabilities that can cope with the challenges of an intelligent environment.

Furthermore, the findings also had important implications for AI-assisted learning technology developers. Firstly, this study found that between user enjoyment experience and user usage attitude, the latter exerts a greater effect. However, the shaping of user attitude is still overlooked by AI application developers, and very few AI-assisted learning products have been specifically designed to create interactive activities to enhance learners’ motivation, engagement, and attitude and reduce anxiety toward language learning [85]. Developers can indirectly influence users' attitudes towards AI technology by expanding user groups and enhancing user empathy [86]. Secondly, if AI-assisted learning products want to maintain their market share, they also need to pay attention to learners' user experience and satisfaction and reduce the difficulty of technology usage. Moreover, AI-assisted learning products need to carefully consider how to empower learners, enhance their self-efficacy in learning, and try to reduce the negative impact of technical difficulties [87,88]. Finally, learners should not overly exaggerate the difficulties of AI-assisted learning scenarios when participating in online English learning. They should fully recognize that perseverance and interest in learning are key to achieving learning expectations and persisting in AI-assisted EFL learning activities [89].

## 6 Conclusion

This study employed structural equation modeling to explore the predictive role of learners' AI literacy on their continuance intention for AI-assisted EFL learning, as well as the mediating effects of attitudes towards AI-assisted EFL and foreign language enjoyment. The findings indicated that learners' AI literacy positively and significantly affects their attitudes towards using AI technology for EFL learning, which positively affects foreign language enjoyment. Furthermore, foreign language enjoyment positively impacts the willingness to continue using AI technology for EFL learning, and learners' AI literacy also has a direct effect on this willingness. The attitude towards AI-assisted EFL learning and foreign language enjoyment serve as partial mediating variables between learners' AI literacy and their willingness to continue AI-assisted foreign language learning.

The study does have certain limitations. Firstly, the research subjects are undergraduate students, who represent a relatively small proportion of online English learners in China, limiting the representativeness of the entire college student population. Secondly, learners' many emotional qualities and psychological traits are involved in AI-supported English learning. Future research could attempt to incorporate other positive emotions, such as interest and confidence, into the study to improve the understanding of the mechanism affecting learners' intention to continue AI-assisted EFL learning.

## Ethics approval and consent to participate

All methods followed the guidelines and regulations of the 1964 Helsinki Declaration and its later amendments. Since this study does not involve intervention and is low-risk, ethical review and approval were waived according to the institutional review boards at the School of Foreign Languages at Huainan Normal University.

All participants were required to sign a written informed consent form. They were also informed about the purpose of the study and their right to withdraw from it at any time. All methods were carried out under relevant guidelines and regulations.

## Consent for publication

Not applicable.

## Data Availability statement

Data will be available upon request.

## Funding

This study was funded by the SFLEP World Language and Culture Research Project (grant number: WYZL2023AH0004). The research was supported by the 10.13039/501100017588HNNU teaching team project (grant number:2021hsjxtd04)

## CRediT authorship contribution statement

**Jiqun Fan:** Writing – review & editing, Writing – original draft, Validation, Software, Methodology, Data curation, Conceptualization. **Qinqing Zhang:** Writing – review & editing, Funding acquisition, Data curation, Conceptualization.

## Declaration of competing interest

The authors declare that they have no known competing financial interests or personal relationships that could have appeared to influence the work reported in this paper.

## Figures and Tables

**Table:** 

Construct	Items	Source
AIL	I understand why AI technology needs big data.	[63]
	I know the processes through which deep learning enables AI to perform voice recognition tasks.	
	I understand how AI technology optimizes the translation output for online translation.	
	I know how AI can be used to predict possible outcomes through statistics.	
	I understand how AI assistants such as SIRI or Hello Google handle human-computer interaction.	
	I understand how computers process images to produce visual recognition.	
ATT	AI-assisted EFL learning is a joyful experience.	[65]
	AI-assisted EFL learning provides me with a sense of satisfaction.	
	AI-assisted EFL learning meets my educational needs.	
	AI-assisted EFL learning instills confidence in me.	
	AI-assisted EFL learning brings me a sense of pleasure.	
FLE	I don't get bored.	[64]
	I don't enjoy this term's AI-assisted EFL class.	
	I performed well in this term's AI-assisted EFL class.	
	I don't feel proud of my accomplishments in this term's AI-assisted EFL class.	
	It is a positive environment.	
	It is cool to learn English with AI assistance.	
	AI-assisted EFL class is not fun.	
	My peers in the AI-assisted EFL class are not nice.	
	There is a good atmosphere for AI-assisted EFL learning.	
	We laugh a lot in AI-assisted EFL class.	
CI	If possible, I would continue with AI-assisted EFL learning.	[66]
	I would recommend AI-assisted EFL learning resources to my classmates and friends.	
	I am inclined to continue AI-assisted EFL learning over other alternative forms of learning.	
	I will continue to utilize AI-assisted EFL learning resources in the future.	
