# Supplementary material for: From literacy to learning: The sequential mediation of attitudes and enjoyment in AI-assisted EFL education
Source: Heliyon. 2024 Aug 30;10(17):e37158. doi: 10.1016/j.heliyon.2024.e37158 (PMC11402731; doi:10.1016/j.heliyon.2024.e37158)
Supplement: Multimedia component 1 [file mmc1.docx]

亲爱的同学

您好！

感谢您抽出时间填写本问卷。问卷旨在探索学生在人工智能辅助英语学习过程中人工智能素养，学习者态度和外语愉悦对持续学习意愿的影响。问卷采用匿名的方式搜集，仅用于学术研究，我们承诺对您的问卷内容保密。参与本问卷无任何风险。[如有任何疑问请发邮件至106050@hnnu.edu.cn](mailto:如有任何疑问请发邮件至liuhonggang@suda.edu.cn) 。

如果您同意填写本问卷，请选择继续。如果您不同意填写本问卷，请点击“否”选项，系统将自动终止问卷。

感谢您的支持！

项目负责人：淮南师范学院外国语学院 樊继群

联系邮件：106050@hnnu.edu.cn

电话：15856690505

**一 人口统计学测量项**

D1.您的性别是

1. 男 （ ） 2.女（ ）

D2.您所在年级为

1.一年级 （ ） 2. 二年级 （ ） 3. 三年级（ ） 4. 四年级 （ ）

D3..您的专业属于

1. 自然科学类（ ） 2. 工程技术类（ ） 3. 人文社会科学类（ ）

D4您在外语学习中使用AI技术的目的是：（可多选）

1.外语听说训练（ ） 2. 单词与知识记忆（ ） 3. 智能翻译（ ） 4. 信息搜索和文本生成（ ） 5. 语言测试与评价（ ） 6. 个性化学习规划与监控（ ）

7. 文化理解与兴趣培养（ ）

D5.您每周外语学习使用AI技术的时长（length）是：

1. *从不* （ ） 2. *≤1小时*（ ） 3. *1小时*＜*（length）≤3小时*（ ） 4. *3小时＜（length）≤7小时*（ ） 5. *7小时＜（length）≤10小时*（ ） 6. *10小时＜（length）≤15小时*（ ） 7. *＞15小时*（ ）

D6.你在外语学习中使用AI技术的媒介或环境是：（可多选）

1.手机 （ ） 2.平板 （ ） 3. 笔记本电脑 （ ） 4. 学校实验室设备（ ）

D7.您在外语学习中使用过的AI工具：（可多选）

1.Chatgpt（ ） 2. 文心一言（ ） 3. 质谱清言（ ）4.讯飞星火（ ） 5. 通义千问（ ） 6. 小爱同学（ ） 7. 科大讯飞（ ） 8. Siri（ ）9.小艺（ ） 10.小布助手（ ） 11. 有道词典（ ） 12. 扇贝单词（ ） 13.百词斩（ ） 14.多邻国（ ） 15.腾讯翻译君（ ）

**二 外语学习AI技术使用调查**

**每个陈述后有五个选项，1-5分别是：1“非常不同意”，2“不同意”， 3 “中立”，4“同意”，5“非常同意”。**请您在每个陈述后勾选（✔）一个最符合您意见的选项。

| **题号** | **题项（1：非常不同意，2：不同意，3：中立，4：同意，5：非常同意）** | **1** | **2** | **3** | **4** | **5** |
| --- | --- | --- | --- | --- | --- | --- |
| 1 | 我知道为什么AI技术需要大数据。 |  |  |  |  |  |
| 2 | 我了解深度学习时AI技术会执行语音识别任务。 |  |  |  |  |  |
| 3 | 我了解AI技术可以优化在线翻译。 |  |  |  |  |  |
| 4 | AI助手如“你好，谷歌”、“SIRI”等可实现人机互动。 |  |  |  |  |  |
| 5 | 我知道AI技术可以根据数据预测可能的结果。 |  |  |  |  |  |
| 6 | 我知道计算机如何处理图像以进行视觉识别。 |  |  |  |  |  |
| 7 | AI辅助外语学习是一个快乐的体验。 |  |  |  |  |  |
| 8 | AI辅助外语学习让我有一种满足感。 |  |  |  |  |  |
| 9 | AI辅助外语学习可以满足我的教育需求。 |  |  |  |  |  |
| 10 | AI辅助外语学习让我信心满满。 |  |  |  |  |  |
| 11 | AI辅助外语学习给我带来快乐感。 |  |  |  |  |  |
| 12 | 我不觉得无聊。 |  |  |  |  |  |
| 13 | 我不喜欢本学期的外语听说课。 |  |  |  |  |  |
| 14 | 我在AI技术支持的外语课程上总体表现很好。 |  |  |  |  |  |
| 15 | 我不会为自己在AI技术支持的外语课程上取得的成绩感到骄傲。 |  |  |  |  |  |
| 16 | AI技术支持的外语课堂氛围很积极。 |  |  |  |  |  |
| 17 | 使用AI学外语很酷。 |  |  |  |  |  |
| 18 | AI技术支持的外语课程没意思 。 |  |  |  |  |  |
| 19 | 在AI技术支持的外语课上同学并不好。 |  |  |  |  |  |
| 20 | AI技术支持的外语课程有良好的课堂氛围。 |  |  |  |  |  |
| 21 | 我们在AI技术支持的外语课程上很快乐。 |  |  |  |  |  |
| 22 | 如有可能，我会继续使用AI工具辅助外语学习。 |  |  |  |  |  |
| 23 | 我会向朋友、同学推荐使用AI工具辅助外语教学。 |  |  |  |  |  |
| 24 | 我有意继续使用AI工具辅助外语学习而非其他替代学习方式。 |  |  |  |  |  |
| 25 | 将来我仍会使用AI工具辅助外语学习。 |  |  |  |  |  |
|  | | | | | | |

感谢您的支持与参与！
